# ApoE influences regional white-matter axonal density loss in Alzheimer's disease

**DOI:** 10.1016/j.neurobiolaging.2017.04.021

**Published:** 2017-09

**Authors:** Catherine F. Slattery, Jiaying Zhang, Ross W. Paterson, Alexander J.M. Foulkes, Amelia Carton, Kirsty Macpherson, Laura Mancini, David L. Thomas, Marc Modat, Nicolas Toussaint, David M. Cash, John S. Thornton, Susie M.D. Henley, Sebastian J. Crutch, Daniel C. Alexander, Sebastien Ourselin, Nick C. Fox, Hui Zhang, Jonathan M. Schott

**Affiliations:** a Department of Neurodegenerative Disease, Institute of Neurology, UCL, London, UK; b Department of Computer Science and Centre for Medical Image Computing, UCL, London, UK; c Neuroradiological Academic Unit, Department of Brain Repair and Rehabilitation, UCL Institute of Neurology, London, UK; d Lysholm Department of Neuroradiology, National Hospital for Neurology and Neurosurgery, UCLH NHS Foundation Trust, London, UK; e Leonard Wolfson Experimental Neurology Centre, UCL Institute of Neurology, London, UK; f Translational Imaging Group, Centre for Medical Image Computing, Department of Medical Physics and Biomedical Engineering, UCL, London, UK

**Keywords:** Alzheimer's disease, Diffusion tensor imaging, Neurite orientation dispersion and density imaging, Apoe, White matter, Phenotype

## Abstract

Mechanisms underlying phenotypic heterogeneity in young onset Alzheimer disease (YOAD) are poorly understood. We used diffusion tensor imaging and neurite orientation dispersion and density imaging (NODDI) with tract-based spatial statistics to investigate apolipoprotein (APOE) ε4 modulation of white-matter damage in 37 patients with YOAD (22, 59% APOE ε4 positive) and 23 age-matched controls. Correlation between neurite density index (NDI) and neuropsychological performance was assessed in 4 white-matter regions of interest. White-matter disruption was more widespread in ε4+ individuals but more focal (posterior predominant) in the absence of an ε4 allele. NODDI metrics indicate fractional anisotropy changes are underpinned by combinations of axonal loss and morphological change. Regional NDI in parieto-occipital white matter correlated with visual object and spatial perception battery performance (right and left, both *p* = 0.02), and performance (nonverbal) intelligence (WASI matrices, right, *p* = 0.04). NODDI provides tissue-specific microstructural metrics of white-matter tract damage in YOAD, including NDI which correlates with focal cognitive deficits, and APOEε4 status is associated with different patterns of white-matter neurodegeneration.

## 1 Introduction

The biological underpinnings of phenotypic expression in neurodegenerative disease are poorly understood. Clinical heterogeneity is particularly apparent in patients with sporadic young onset AD (YOAD), defined as symptom onset at less than 65 years (Rossor et al., 2010). These individuals are more likely to present with nonamnestic phenotypes, including posterior cortical atrophy (Tang-Wai et al., 2004), logopenic progressive aphasia (Gorno-Tempini et al., 2011) and a dysexecutive/behavioural syndrome (Ossenkoppele et al., 2015b). Although the distribution of Aβ plaques is broadly similar across these atypical phenotypes (Lehmann et al., 2013), there are marked differences in the focality of tau tangle pathology (Ossenkoppele et al., 2016) and brain atrophy (Ossenkoppele et al., 2015a), both of which correlate with symptoms (Ossenkoppele et al., 2015a, Ossenkoppele et al., 2016).

Factors initiating and potentiating this selective vulnerability and differential expression of pathology across the brain are likely to be driven, at least in part, by genetic influences. Apolipoprotein (APOE) ε4 is the most important genetic risk factor for sporadic AD (Strittmatter and Roses, 1995). In late onset AD (LOAD), APOE ε4 reduces the age of onset (Corder et al., 1993), is associated with medial temporal lobe atrophy (Filippini et al., 2009), and tends to manifest as a ‘typical’ amnestic phenotype (van der Vlies et al., 2007). However, evidence suggests that patients with atypical YOAD are less likely to carry an APOE ε4 allele (van der Flier et al., 2011), which may account for some of the differences in structural damage and clinical presentation.

**Fig. 1:**
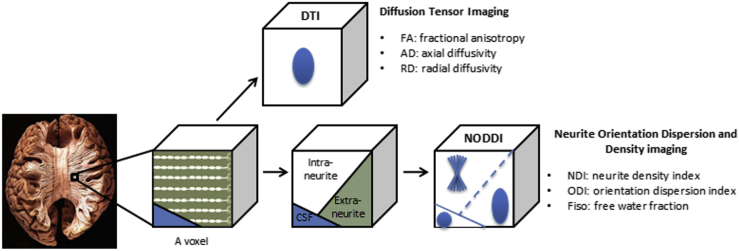
Diffusion tensor imaging (DTI) and neurite orientation dispersion and density imaging (NODDI) models for diffusion-weighted MRI. DTI models each voxel using a single tensor, hence gives a composite view of tissue microstructure. NODDI models each voxel as 3 compartments: intraneurite (restricted diffusion), extraneurite (hindered diffusion), and cerebrospinal fluid (isotropic diffusion). Dendrites and axons, collectively known as ‘neurites’, are projections of neurons. NODDI can estimate neurite density index (NDI) and orientation dispersion index (ODI), specifically in the intraneurite compartment, without partial volume effects from free water.

From a mechanistic perspective, a potential unifying explanation for the mismatch between widespread amyloid deposition and more focal downstream neurodegeneration comes from prion-like spread of proteinopathies with tropism for specific large scale neural networks (Seeley et al., 2009, Warren et al., 2013). Diffusion tensor imaging (DTI) is a magnetic resonance (MR) technique for exploring the structural integrity of white-matter in vivo using water diffusion to distinguish different microstructural environments. DTI provides voxel-level estimates of fractional anisotropy (FA), axial diffusivity (AxD), and radial diffusivity (RD). Neurite orientation dispersion and density imaging (NODDI; Zhang et al., 2012) is one of a number of advanced diffusion MRI techniques designed to probe tissue microstructure beyond a composite view of each voxel by modeling water diffusion in multiple compartments (Ferizi et al., 2014), that is, diffusion that is restricted in axons and dendrites, hindered in extraneurite space and isotropic in cerebrospinal fluid. NODDI derives a neurite density index (NDI), orientation dispersion index (ODI), and the fraction of free water (F_iso_; Fig. 1). This model allows axonal loss in white matter (NDI) to be distinguished from altered patterns of axonal organization (ODI) on a voxel-by-voxel basis, thereby disentangling 2 key factors contributing to changes in FA. Similar to DTI, NODDI can be implemented with standard diffusion-weighted imaging sequences making it accessible for routine clinical studies. Its utility has been widely demonstrated in a broad range of applications, including Parkinson's disease (Kamagata et al., 2016), epilepsy (Winston et al., 2014), normal aging (Billiet et al., 2015), brain development (Kunz et al., 2014), and neurogenetic disorders (Timmers et al., 2015).

In this study, we apply DTI and NODDI to a population of patients with YOAD and healthy controls to investigate the nature of microstructural damage underpinning changes in FA and test the hypotheses that (1) APOE ε4 status modulates regional signatures of white-matter network breakdown and (2) reduction in white-matter NDI, reflecting neurite loss, influences the clinical phenotype of YOAD.

## 2 Methods and materials

### 2.1 Study participants

Forty-five patients meeting consensus criteria for probable AD (McKhann et al., 2011) with symptom onset <65 years were recruited prospectively from 2013 to 2015 from a specialist Cognitive Disorders clinic. None had a known mutation or family history suggestive of autosomal dominant inheritance. The presenting cognitive symptom was recorded for all patients, and patients were classified as having a typical (McKhann et al., 2011) or atypical (posterior cortical atrophy [Tang-Wai et al., 2004]) AD phenotype according to published criteria. Twenty-four healthy controls matched for mean age and gender were recruited. All participants underwent MRI scanning, testing on the Mini–Mental State Examination (MMSE; Folstein et al., 1975), assessment on the Hachinski Ischaemic Score (Moroney et al., 1997), and an extensive neuropsychology battery designed to capture phenotypic diversity in YOAD including: assessment of general intellect (vocabulary and matrices subtests of the Wechsler Abbreviated Scale of Intelligence, WASI [Wechsler, 1999]), episodic memory for faces and words (Recognition Memory Test, RMT [Warrington, 1984]), numeracy, and literacy (Graded Difficulty Arithmetic, GDA [Jackson and Warrington, 1986]; Graded Difficulty Spelling Test, GDST [Baxter and Warrington, 1994]), visuospatial, and visuoperceptual performance (Visual Object and Spatial Perception battery, VOSP [Warrington and James, 1991]), speed of processing and executive function (DKEFS, Delis-Kaplan Executive Function System–design fluency test, DKEFS (Delis et al., 2001); Digit Symbol Modalities Test, DSMT (Smith, 1986); verbal fluency). Ethical approval was obtained from the National Hospital for Neurology and Neurosurgery Research Ethics Committee and written informed consent obtained from all the participants.

### 2.2 APOE genotyping

Patient participants gave separate specific consent to donate blood for genetic analyses. DNA was extracted and *APOE* genotype was determined by PCR with 3′-minor groove binding probes.

### 2.3 MRI acquisition

MRI was performed on a Siemens Magnetom Trio (Siemens, Erlangen, Germany) 3T MRI scanner using a 32-channel phased array head coil. Two identical diffusion-weighted imaging acquisitions were performed using a single-shot, spin-echo echo planar imaging sequence (64 diffusion-weighted directions, b = 1000 s/mm^2^; 9 b = 0 s/mm^2^ images (referred to as ‘b0’ images); 55 slices; voxel size 2.5 × 2.5 × 2.5 mm^3^; TR/TE = 6900/91 ms; total acquisition time for both sequences = 16:29 minutes). A 3-shell diffusion sequence optimized for NODDI was acquired (64, 32, and 8 diffusion-weighted directions at b = 2000, 700, and 300 s/mm^2^; 14 b = 0 images; 55 slices; voxel size 2.5 × 2.5 × 2.5 mm^3^; TR/TE = 7000/92 ms; total acquisition time = 16:13 minutes). Both single-shell (DTI) and multishell (NODDI) diffusion-weighted sequences utilize twice-refocused spin echo to minimize distortion effects from eddy-currents. All scans were assessed for quality control purposes, based on coverage and movement artifact.

### 2.4 DTI and NODDI image processing

Sixty participants (37 YOAD, 23 controls) had both DTI and NODDI data that passed quality control criteria and were included for analysis. Images were confirmed to have minimal eddy-current distortion and were corrected for motion by rigidly registering each diffusion-weighted image to the first b0 image using FLIRT (Jenkinson and Smith, 2001, Jenkinson et al., 2002). Diffusion tensor volumes were spatially normalized with DTI-TK (http://dti-tk.sourceforge.net) which bootstraps a population-specific tensor template from the input tensor volumes and aligns them to the template in an iterative fashion (Zhang et al., 2007) with a tensor-based registration algorithm (Zhang et al., 2006). This framework has been shown to improve white-matter alignment compared with conventional FA-based registration (Wang et al., 2011). DTI metrics (FA, AxD, RD) were estimated using FSL (Jenkinson et al., 2012). NODDI metrics (NDI, ODI, F_iso_) were estimated using the NODDI Matlab toolbox (http://www.nitrc.org/projects/noddi_toolbox).

### 2.5 Statistical analyses

Patient neuropsychology raw scores (x) were converted to z scores using the formula z = (x − μ)/σ (σ—control population standard deviation, μ—control population mean). Mean z scores were calculated for each neuropsychological test within participant groups, and across neuropsychological tests (where applicable) to generate a composite score for each cognitive domain (Table 1). Statistical tests comparing clinical characteristics and neuropsychology scores were performed in Stata version 12.

**Table 1:** Study participants' demographic, neuropsychological, and clinical characteristics

	Controls n = 23		YOAD n = 37		*p*	APOE ε4− n = 15		APOE ε4+ n = 22		*p*
	Mean	SD	Mean	SD		Mean	SD	Mean	SD	
Demographic and clinical										
Sex, M:F, n	10:13	-	18:19	-	0.8^a^	7:8	-	11:11	-	1.0^a^
Age (y)	60.7	6.0	62.3	4.9	0.3^b^	60.2	3.8	63.6	5.2	0.03^b^
Handedness, L:R, n	3:20	-	1:36	-	0.2^a^	0:15	-	1:21	-	1.0^a^
Years of education	16.7	3.1	14.9	2.8	0.03^a^	15.5	2.3	14.5	3.0	0.3^a^
MMSE (/30)	29.3	1.0	21.3	4.5	<0.0001^c^	19.9	4.4	22.3	4.4	0.1^c^
Age at onset (y)	-	-	56.8	4.4	n/a	55.4	4.3	57.8	4.4	0.1^b^
Disease duration (y)	-	-	5.4	3.2	n/a	4.8	3.0	5.9	3.3	0.3^b^
Neuropsychology										
General intellect: verbal IQ (WASI vocabulary), z score	-	-	−1.15	1.9	-	−1.56	2.3	−0.88	1.6	0.4^c^
General intellect: performance IQ (WASI matrices), z score	-	-	−**5.80**	2.4	-	−**6.24**	2.3	−**5.43**	2.39	0.4^c^
Episodic memory for faces (RMT), z score	-	-	−1.90	1.8	-	−1.65	1.3	−**2.08**	2.1	0.6^c^
Episodic memory for words (RMT), z score	-	-	−**4.35**	2.6	-	−**4.38**	2.7	−**4.33**	2.5	0.8^c^
Literacy and numeracy (GDST, GDA), z score	-	-	−**2.04**	1.3	-	−**2.54**	1.0	−1.70	1.4	**0.04**^b^
Visuoperceptual and visuospatial (VOSP), z score	-	-	−**6.81**	6.5	-	−**8.34**	5.7	−**5.76**	7.0	0.09^c^
Speed of processing and executive function (DKEFS, verbal fluency, DSMT), z score	-	-	−**2.23**	0.8	-	−**2.6**	0.7	−**1.97**	0.7	**0.01**^c^
Phenotype	n		n			n		n		
Leading symptom, memory/visual/language/behavioural	n/a		24/13/0/0		n/a	8/7/0/0		16/6/0/0		0.3^a^

Neuropsychology scores shown are mean z scores for each cognitive domain (a z score <−1.96 indicates statistical difference from controls at *p* < 0.05, indicated in bold). Probability values for neuropsychology scores show significance value comparing APOE ε4− and APOE ε4+ patient groups.

Key: APOE, apolipoprotein E; DKEFS, Delis-Kaplan Executive Function System (design fluency test); DSMT, Digit Symbol Modalities Test; F, female; GDA, Graded Difficulty Arithmetic; GDST, Graded Difficulty Spelling Test; IQ, intelligence quotient; M, male; MMSE, Mini–Mental State Examination; n, number; neg, negative; *p*, probability; pos, positive; RMT, Recognition Memory Test; SD, standard deviation; VOSP, Visual Object and Spatial Perception battery; WASI, Wechsler Abbreviated Scale of Intelligence; YOAD, young onset Alzheimer's disease.

a Two-sided Fisher's exact.

b Two-tailed *t*-test.

c Wilcoxon rank sum.

The Tract-Based Spatial Statistics (Smith et al., 2006) pipeline from FSL (Smith et al., 2004), optimized (Bach et al., 2014) by incorporating a population-specific template (Keihaninejad et al., 2012) with tensor-based registration (Keihaninejad et al., 2013, Zhang et al., 2006) as described in 2.4, was used to detect whole-brain white-matter differences between YOAD groups as defined by APOE ε4 status relative to controls, including age and gender as covariates (5000 permutations, corrected for multiple comparisons with Threshold-Free Cluster Enhancement [Winkler et al., 2014], *p* < 0.05).

To assess the relationship between microstructural tissue changes and clinical phenotype in YOAD patients, correlations between NDI, ODI, and F_iso_ with neuropsychological performance (z scores by cognitive domain) were assessed in 4 manually-defined regions of interest corresponding to left and right posterior quadrants (parieto-occipital lobe projections) and left and right anterior quadrants (fronto-temporal lobe projections) of the mean white-matter skeleton. These ROIs were delineated by dividing the white-matter skeleton into 4 areas at coordinates (x = 112, y = 88) in standard template space. Mean NODDI metrics (NDI, ODI, and F_iso_) within each quadrant ROI were calculated for each individual, and for patient and control groups. Age and gender were included as covariates and correlations with a *p* < 0.05 were reported.

## 3 Results

### 3.1 Demographics, clinical and genetic characteristics

Demographic and clinical data for participant groups are summarized in Table 1. Mean age and gender did not differ significantly between patients and controls (age, *p* = 0.3; gender, *p* = 0.8), and no individual scored >4 on the Hachinski Ischaemic Score. APOE ε4 status was available for the 37 YOAD patients, of whom 22 (59%) were APOE ε4 positive (18 heterozygotes, 4 homozygotes). Patients who had an APOE ε4 allele (ε4+) were significantly older than those who did not (ε4 negative patients, ε4−) at enrollment to the study (*p* = 0.03). There were no significant differences between ε4+ and ε4− patients for age at clinical symptom onset, clinical disease duration at enrollment to the study, or MMSE. The majority of ε4+ patients presented with a ‘typical AD’ amnestic phenotype (16/22, 73%; 3/4, 75% of the ε4+ homozygotes), whereas the ε4− patients contained approximately equal numbers of individuals with an amnestic (8/15, 53%) and atypical visual-led posterior cortical atrophy presentations (7/15, 47%); or alternatively expressed 67% of the amnestic patients carried one or more ε4 alleles, whereas only 46% of the nonamnestic patients were ε4+ve.

### 3.2 Neuropsychological profiles

Neuropsychological analyses showed that as expected, patients with YOAD had multidomain cognitive impairment, performing significantly less well than controls on measures of performance IQ; recognition memory for words; literacy and numeracy; visuospatial and visuoperceptual performance; speed of information processing and executive function (Table 1). ε4− patients were more impaired on tests of literacy and numeracy (*p* = 0.04) and speed of information processing and executive function (*p* = 0.01) than ε4+ positive patients, despite there being no significant difference in the clinical disease durations between the patient groups.

### 3.3 White-matter structural differences by APOE ε4 genotype

**Fig. 2:**
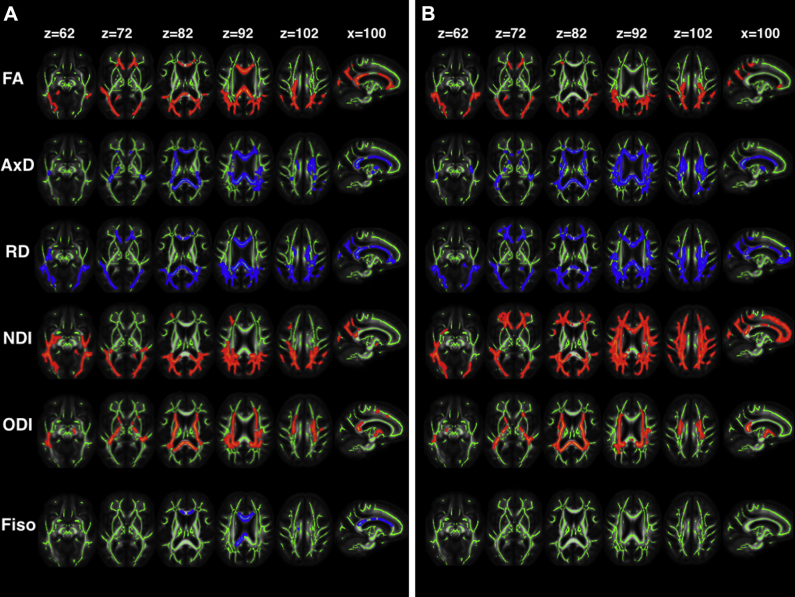
DTI and NODDI metrics in patients with (A) ε4− YOAD (n15) and (B) ε4+ YOAD (n = 22) relative to controls (n = 23). Voxel-wise group differences are shown in red for metrics that are decreased in patients and blue for those increased in patients. Results are overlaid on axial and sagittal sections of the group-specific white-matter skeleton (shown in green) in neurologic convention (the left side appears on the left). Abbreviations: AxD, axial diffusivity; FA, fractional anisotropy; F_iso_, fraction of isotropic water; L, left; NDI, neurite density index; ODI, orientation dispersion index; R, right; RD, radial diffusivity; YOAD, young onset Alzheimer's disease. (For interpretation of the references to color in this figure legend, the reader is referred to the Web version of this article.)

DTI and NODDI metrics are shown for ε4− and ε4+ patients relative to controls in Fig. 2. Both patient groups had decreased FA in white-matter tracts projecting from the parieto-occipital lobes (inferior longitudinal fasciculus, inferior fronto-occipital fasciculus, superior longitudinal fasciculus, genu of corpus callosum, posterior thalamic radiation), with ε4− patients also having decreased FA in the splenium of the corpus callosum and anterior corona radiata. AxD and RD were increased in both patient groups relative to controls in the genu, body and splenium of the corpus callosum, and parieto-occipital white-matter projections (those listed above and the internal capsules). ε4+ patients additionally had increased RD in the white-matter projections from the frontal lobes. There were no areas where patients had increased FA or decreased diffusivity relative to controls, and no significant differences in any DTI metric when APOE ε4+ and ε4− groups were compared directly.

**Fig. 3:**
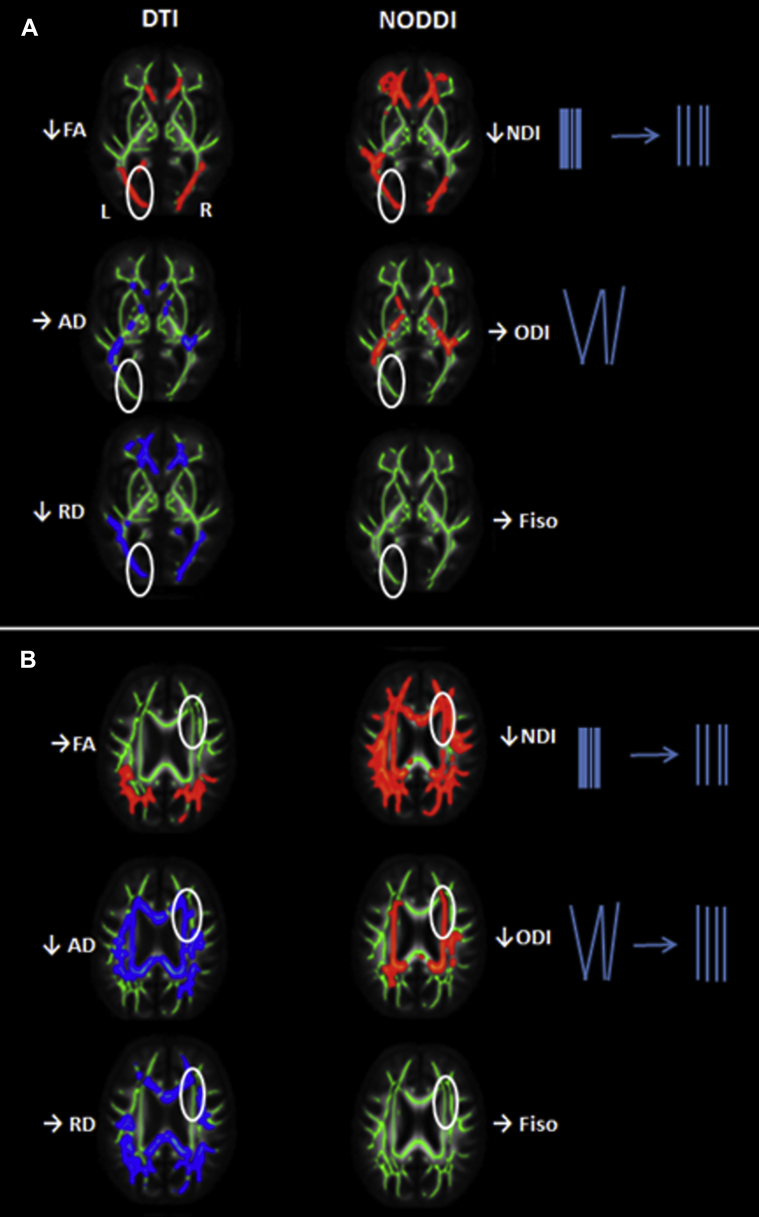
Added sensitivity and specificity of NODDI over DTI. (A) Left posterior white microstructural changes in ε4+ (n = 22) relative to controls (n = 23). Patients with ε4+ YOAD have lower FA and increased RD. NODDI metrics for this region suggest that the underlying microstructural change is decreased neurite density, rather than alteration in neurite orientation, illustrating the additional specificity of NODDI. (B) Right frontal white microstructural changes in ε4+ (n = 22) relative to controls (n = 23). There is no significant change in FA. However, AD increases suggesting underlying microstructural damage, which is corroborated by NODDI metrics revealing reduction in both NDI and ODI (which would tend to affect FA in opposite directions and hence manifest as no overall change in FA). Here, NODDI metrics are more sensitive than FA by avoiding canceling effects. Abbreviations: AxD, axial diffusivity; FA, fractional anisotropy; L, left; NDI, neurite density index; NODDI, neurite orientation dispersion and density imaging; ODI, orientation dispersion index, R, right; RD, radial diffusivity.

The tissue specificity afforded by NODDI revealed a landscape of microstructural damage in YOAD underpinning these changes in DTI metrics; for example, identifying areas of FA reduction specifically due to decreased NDI rather than changes in ODI (Fig. 3A). NODDI metrics could also be more sensitive to change; for example, identifying regions where NDI and ODI reduction occurred in parallel, hence resulting in no overall FA change (Fig. 3B).

**Fig. 4:**
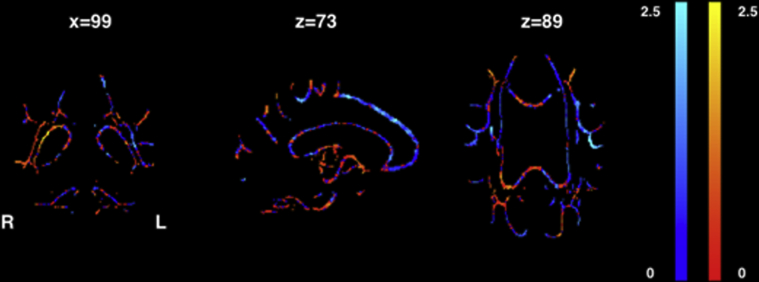
Coronal (left), sagittal (middle), and axial (right) neurite density index t-statistic maps showing areas where APOE ε4− patients have reduced NDI relative to ε4+ patients (warm colors) and where ε4+ patients have reduced NDI relative to ε4− patients (cool colors). (For interpretation of the references to color in this figure legend, the reader is referred to the Web version of this article.)

NDI was reduced in the parieto-occipital white-matter projections (same as listed above) of both ε4− and ε4+ patient groups relative to controls but was strikingly more widespread in ε4+ patients, additionally affecting the body and genu of the corpus callosum, and extending further into the frontal and temporal lobe white-matter projections. ε4− and ε4+ patient groups had a common signature of decreased ODI in the posterior parts of the corpus callosum and internal capsule. ε4− patients also had increased F_iso_ in the corpus callosum, whereas there were no significant differences in F_iso_ in ε4+ patients relative to controls. There were no significant differences in any NODDI metric when directly comparing ε4− and ε4+ patient groups at the prescribed statistical threshold; however plotting the NDI t-statistic maps (uncorrected; Fig. 4) revealed that ε4− patients had the greatest NDI reduction relative to ε4+ in the right parietal lobe white-matter projections; and ε4+ patients had greatest reduction in NDI relative to ε4− in the left temporal lobe projections.

### 3.4 Region of interest NDI correlations with cognitive function

**Fig. 5:**
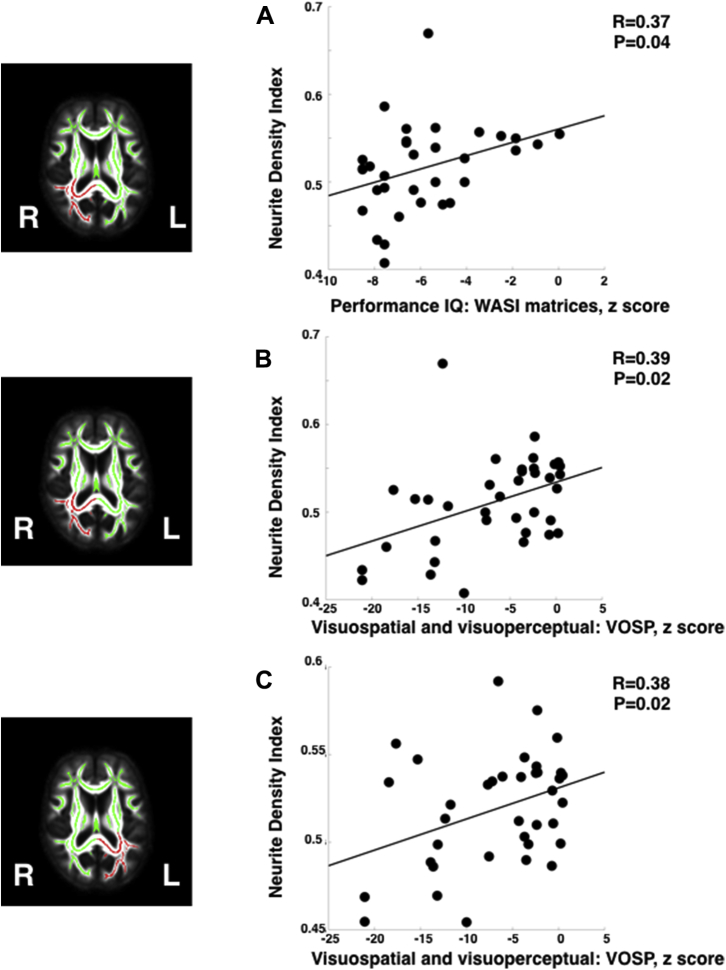
Significant correlations between regional neurite density index and neuropsychological measures in white-matter projections from the right (A and B) and left (C) parieto-occipital cortices of patients with YOAD (n = 37). Regions of interest (red) are shown on the group mean white-matter skeleton (green) to the left of each graph. Abbreviations: IQ, intelligence quotient; L, left; R, right; VOSP, visual object and spatial perception battery; WASI, Wechsler Abbreviated Scale of Intelligence. (For interpretation of the references to color in this figure legend, the reader is referred to the Web version of this article.)

Raw values for NODDI metrics in each of the 4 regions of interest are shown in Table 2. There were significant differences between patients and control NDI metrics in each of the 4 quadrants. In patients, there were significant positive correlations between a visually-demanding measure of performance IQ (WASI matrices) and regional NDI in white-matter projections from the right parieto-occipital lobe (Fig. 5A) and between visuospatial and visuoperceptual tasks and NDI in white-matter projections from the parieto-occipital lobes bilaterally (Fig. 5B and C). There were no significant positive correlations between NDI and performance on other cognitive domains, and no significant negative correlations between regional NDI and any cognitive score. Regional ODI metrics did not correlate with any cognitive score. F_iso_ in the left posterior quadrant correlated with patient episodic memory for words (r = 0.4, *p* = 0.01).

**Table 2:** NODDI metrics in each region of interest

WM ROIs	YOAD	Controls	*p*^a^
NDI (mean ± SD)			
Left anterior quadrant	0.554 ± 0.022	0.538 ± 0.031	0.043
Left posterior quadrant	0.559 ± 0.029	0.518 ± 0.031	<0.00002
Right anterior quadrant	0.555 ± 0.022	0.539 ± 0.036	0.067
Right posterior quadrant	0.552 ± 0.029	0.511 ± 0.052	0.001
ODI (mean ± SD)			
Left anterior quadrant	0.214 ± 0.015	0.214 ± 0.007	0.95
Left posterior quadrant	0.202 ± 0.023	0.201 ± 0.007	0.76
Right anterior quadrant	0.213 ± 0.014	0.211 ± 0.008	0.54
Right posterior quadrant	0.195 ± 0.013	0.194 ± 0.008	0.68
F_iso_ (mean ± SD)			
Left anterior quadrant	0.105 ± 0.017	0.108 ± 0.016	0.67
Left posterior quadrant	0.107 ± 0.024	0.111 ± 0.017	0.58
Right anterior quadrant	0.099 ± 0.017	0.106 ± 0.018	0.22
Right posterior quadrant	0.101 ± 0.023	0.111 ± 0.034	0.26

Key: F_iso_, fraction of free water; NDI, neurite density index; NODDI, neurite orientation dispersion and density imaging; ODI, orientation dispersion index; *p*, probability; ROI, region of interest; SD, standard deviation; WM, white matter; YOAD, young onset Alzheimer's disease.

a Two-tailed *t*-test.

## 4 Discussion

In this study, we used DTI and NODDI to explore signatures of white-matter structural brain damage and their biological underpinnings in patients with YOAD on the basis of APOE ε4 genotype. DTI metrics showed regions of altered white-matter microarchitecture in both ε4+ and ε4− patients relative to controls. NODDI, a multishell diffusion technique implemented on standard 3T clinical MR scanners, provided further insights into the commonalities and differences in white-matter change associated with ε4 genotype; namely more widespread NDI reduction in ε4+ individuals and more focal posterior reductions in patients without an ε4 allele. Finally, reduction in regional NDI—specifically modeling axonal loss in white matter—correlated with patterns of focal cognitive impairment, suggesting that NODDI metrics not only provide insights into regional white-matter vulnerability but also have relevant clinical correlates.

Despite being the most important risk factor for AD, the effects of APOE ε4 on clinical phenotype white-matter damage are incompletely understood. Our observation that ε4 negative patients are impaired more than ε4 positive patients on neuropsychological tests of literacy and numeracy, and speed of information processing and executive function, is consistent with findings from several previous studies whereby ɛ4− patients were shown to be more impaired in nonmemory cognitive domains (van der Flier et al., 2011). The relatively few previous DTI studies in YOAD have shown decreased white-matter FA and increased diffusivity but have focused on describing regional variation between phenotypes (Caso et al., 2015, Cerami et al., 2015, Madhavan et al., 2016) or in YOAD relative to LOAD (Canu et al., 2013) rather than investigating potential differential effects of APOE ε4. Studies in LOAD have shown contradictory findings: APOE ε4 allele status was associated with an increase in parahippocampal white-matter mean diffusivity (Wang et al., 2012), yet Kljajevic et al. found ε4 status affected mean diffusivity in controls, but not in participants with clinically-manifest AD (Kljajevic et al., 2014). Our observation that axial and radial diffusivity changes are more prominent than FA in both the presence and absence of an ε4 allele is consistent with previous observations that these directional diffusivity metrics can be a more sensitive marker of structural change than FA (Acosta-Cabronero et al., 2010). The areas of increased RD and AxD, and FA reduction in bilateral parietal lobes, genu of the corpus callosum, frontal white-matter lobe projections, we report are broadly in keeping with changes in DTI metrics reported in YOAD patients (ε4 status unspecified) previously (Canu et al., 2013), and we additionally show that ε4 status appears to have a modulating effect: less anterior FA reduction in the presence of an ε4 allele. Our NODDI results allow more specific inferences about the nature of the underlying microstructural damage. Thus, as illustrated in Fig. 3. NODDI metrics can explain different mechanisms underlying changes in FA; or indeed detect the effects of concomitant pathological processes that would individually affect FA in opposing ways and hence cancel one another out, resulting in no observable FA change.

White-matter NDI reduction was more extensive anteriorly in ε4+ than ε4− YOAD patients (Fig. 2). Although differences did not survive the statistical threshold for multiple comparisons when comparing the patient groups directly, t-statistic maps (uncorrected) reveal subtle differences in regional NDI values between the ε4− and ε4+ groups (Fig. 4). The former had more NDI loss in right parietal lobe white-matter connections, in keeping with a trend for worse performance on visual tasks. Conversely, the ε4+ group had more NDI reduction in left temporal lobe connections. The similarities and differences are consistent with there being a “generic” signature of network breakdown in AD, with relatively subtle ε4 modulation of network dysfunction, perhaps influencing phenotype through differential propagation of pathology.

Histological evidence supports a relationship between MRI estimates of axonal density reduction and actual axon loss. *Ex vivo* studies in animals have demonstrated that diffusion MRI estimates of axon density, using a related diffusion model, show a high degree of correlation with optical staining intensity of myelin and stereological estimates of axonal volume fraction in white matter (Jespersen et al., 2010). If reduced NODDI, NDI truly reflects axon loss in YOAD, then it follows that even partial disconnection of brain regions should lead to functional consequences that manifest in the phenotype. Although DTI metrics do not correspond to compartment-specific microstructural changes, previous studies have shown correlation with global measures of cognition. In patients with LOAD, fractional anisotropy in the corpus callosum has been shown to correlate with performance on the MMSE (Canu et al., 2013), and more specifically, radial diffusivity and fractional anisotropy in the splenium correlate with dementia severity on the ACE-R (Acosta-Cabronero et al., 2012). In YOAD, global cognitive performance on the Clinical Dementia Rating Sum of Boxes has been found to correlate with mean diffusivity in several brain regions including the corpus callosum, posterior cingulate, frontal and parietal parts of the superior longitudinal fasciculus bilaterally, and left temporal regions (Canu et al., 2013). However, no correlation has been demonstrated between diffusion MRI metrics and performance on focal cognitive test scores sensitive to regional brain dysfunction. We found that NDI in white-matter projections from both left and right parieto-occipital lobes correlated with visuospatial and visuoperceptive cognitive performance, a sensitive marker of nondominant parietal cortex function; bilateral correlations are likely to reflect that the parietal lobes are structurally closely interconnected. Right parieto-occipital white-matter NDI also correlated with a measure of performance (nonverbal) intelligence, reflecting right hemisphere dysfunction. These correlations suggest that regional reductions in NDI can provide in vivo measures of cell loss and network breakdown, which in turn shape clinical phenotype. The lack of correlation of NDI with other neuropsychological scores may reflect these cognitive functions being underpinned by a more distributed white-matter network or relate to the dispersion of results on psychology testing—the tests that showed correlation were those with the largest range of patient performance. The finding of a positive correlation between F_iso_ (i.e., increased water content) in the left posterior quadrant and better episodic memory for words in the patients was unexpected. Unlike neurite density which would be expected to closely relate to brain function and showed differences between patients and controls, the significance of free water—which did not show differences between patients and controls, is far from clear, and we suspect may be a false-positive finding.

Orientation dispersion relates to axonal organization (Jespersen et al., 2012) and white-matter ODI has been reported to increase in normal aging (Billiet et al., 2015). We found ODI to be reduced (i.e., the tracts were more coherent) in the corpus callosum and internal capsule of individuals in both ε4− and ε4+ YOAD patients relative to controls, notably even in some regions unaffected by significant NDI change. The anatomical dissociation may suggest alterations in NDI and ODI reflect different pathophysiological phenomena in neurodegeneration. One possible histological explanation is that reduced orientation dispersion reflects loss of secondary crossing fibers to leave more aligned neurons in the primary tracts, perhaps mediated by selective axonal degeneration. However, the lack of correlation between regional ODI with neuropsychological scores suggests that neurite density may be more critical for cognitive function.

This study has a number of limitations. As with all biophysical models, and specifically multi-compartment diffusion models, NODDI requires a number of assumptions: modeling axon orientation does not account for crossing fibers, the value of intrinsic diffusivity is fixed over the whole brain, and intraneurite diffusion is modeled as being completely restricted within a collection of impermeable sticks. It is conceivable that this does not fully characterize all pathological processes involved in neurodegenerative diseases (e.g., AD) such as possible regional variation in intrinsic diffusivity, alterations in neurite membrane permeability or damage to intraneurite architecture. Jesperson et al. have used a related multi-compartment diffusion model (Jespersen et al., 2007) to examine the relationship between neurite density and orientation dispersion with histological measures in animal brains (Jespersen et al., 2010). There is currently limited histopathological evidence to specifically validate NODDI model metrics in the human brain; however an ex vivo study of human spinal cord in multiple sclerosis showed NODDI replicated the trends of histological indices and could detect specific features of tissue pathology (Grussu et al., 2015).

We also do not account for the potential presence of white-matter hyperintensities (WMHs) which have been shown to result in lower FA and higher MD relative to normal appearing white matter (Maniega et al., 2015). In AD, WMH may be related to coexistent vascular burden, or be part of AD pathogenesis. One of the advantages of studying patients with YOAD is that they are less likely to have significant coexistent vascular disease than individuals with late onset disease, and all participants in this study scored 4 or less on the Hachinski Ischaemic score indicating that on clinical grounds there was not a significant vascular component to their syndrome. There may be more amyloid angiopathy observed in patients with ε4 positive disease than those who do not carry an ε4 allele (Shinohara et al., 2016) which could both explain some of the differences we observed. It is unknown how the presence of different causes of WMH affects NODDI metrics—and indeed future studies with post-mortem verification are needed to see if NODDI may be a useful imaging technique to understand the microstructural changes underpinning these white-matter alterations. If quantifiable, WMH lesions loads could be included as a covariate in the modeling.

The participant groups we report are relatively small, yet indicative of the relative rarity of YOAD and may explain why direct comparisons of ε4+ and ε4− groups were not sufficiently powered to reach statistical significance. Although all patient participants met consensus criteria for probable AD, there was no pathological evidence available to confirm underlying AD pathology. Finally, Alzheimer disease is a complex genetic disease and any modulation of network breakdown due to APOE ε4 here observed likely occurs in the context of attenuation by a host of other genetic and environmental factors.

NODDI metrics in the healthy aging brain show good short interval reproducibility (Chang et al., 2015) but longitudinal studies, ideally with post-mortem histological evaluation, are required to establish if NODDI metrics are robust, reproducible, and capable of tracking Alzheimer's disease progression, characteristics that may give the technique utility in clinical trials. Given the interest in testing potential therapeutic agents at earlier stages of disease, it will be important to assess if—as predicted—NODDI metrics can detect white-matter microstructural changes in people with very mild or pre-symptomatic stages of disease. NODDI could also be applied to studying cortical diffusion in gray matter where microstructural changes may be more sensitive and specific than the macrostructural damage observed as cortical atrophy, and we may expect to see closer correlation between regional NODDI metrics and performance on neuropsychological tests. Finally, in a wider context beyond AD, NODDI may further inform our understanding of network changes in normal aging and other neurodegenerative diseases.

## Disclosures statement

Dr Slattery reports having received personal fees from GE Healthcare. Professor Fox reports personal fees from Janssen/Pfizer, IXICO, Roche, Lilly Research Laboratories (Avid), Novartis Pharma AG, Sanofi and GSK (all fees paid to University College London). The remaining authors declare no conflicts of interest.
